# Engineered living materials for the conversion of a low-cost food-grade precursor to a high-value flavonoid

**DOI:** 10.3389/fbioe.2023.1278062

**Published:** 2023-11-28

**Authors:** Florian Riedel, Maria Puertas Bartolomé, Lara Luana Teruel Enrico, Claudia Fink-Straube, Cao Nguyen Duong, Fabio Gherlone, Ying Huang, Vito Valiante, Aránzazu Del Campo, Shrikrishnan Sankaran

**Affiliations:** ^1^ INM—Leibniz Institute for New Materials, Saarbrücken, Germany; ^2^ Chemistry Department, Saarland University, Saarbrücken, Germany; ^3^ Biobricks of Microbial Natural Product Syntheses, Leibniz Institute for Natural Product Research and Infections Biology—Hans Knöll Institute, Jena, Germany; ^4^ Faculty of Biological Sciences, Friedrich Schiller University Jena, Jena, Germany

**Keywords:** engineered-living-materials (ELMs), flavonoid, pinocembrin, PVA hydrogel, *E. coli* Nissle 1917, probiotic, enzyme catalysis

## Abstract

Microbial biofactories allow the upscaled production of high-value compounds in biotechnological processes. This is particularly advantageous for compounds like flavonoids that promote better health through their antioxidant, anti-bacterial, anti-cancer and other beneficial effects but are produced in small quantities in their natural plant-based hosts. Bacteria like *E. coli* have been genetically modified with enzyme cascades to produce flavonoids like naringenin and pinocembrin from coumaric or cinnamic acid. Despite advancements in yield optimization, the production of these compounds still involves high costs associated with their biosynthesis, purification, storage and transport. An alternative production strategy could involve the direct delivery of the microbial biofactories to the body. In such a strategy, ensuring biocontainment of the engineered microbes in the body and controlling production rates are major challenges. In this study, these two aspects are addressed by developing engineered living materials (ELMs) consisting of probiotic microbial biofactories encapsulated in biocompatible hydrogels. Engineered probiotic *E. coli* Nissle 1917 able to efficiently convert cinnamic acid into pinocembrin were encapsulated in poly(vinyl alcohol)-based hydrogels. The biofactories are contained in the hydrogels for a month and remain metabolically active during this time. Control over production levels is achieved by the containment inside the material, which regulates bacteria growth, and by the amount of cinnamic acid in the medium.

## 1 Introduction

Flavonoids are a group of natural products mainly synthesized by plants found in many foods. Their basic chemical structure is two benzene rings that are linked by a heterocyclic pyran ring. They show a broad range of different biological effects such as antioxidant (Heim et al., 2002), antibacterial (Ohemeng et al., 1993) or anticancer (Wang et al., 2019). Therefore, flavonoids are researched as potential drug candidates for different diseases. In particular, the flavonoid Pinocembrin has been shown to possess therapeutic potential for skin fibrosis and keloid formation based on studies in mice (Li et al., 2021). Furthermore, pinocembrin has been shown to have potential neuroprotective effects for treating Alzheimer’s (Liu et al., 2014) and Parkinson’ disease (Wang et al., 2014), anti-Inflammatory activity (Zhou et al., 2015), antioxidant activity (Shi et al., 2011), antimicrobial activity (e.g., against *Staphylococcus aureus*) (Soromou et al., 2013), vasodilation effects (Li et al., 2013) and hepatoprotection activity (Ma et al., 2023). However, the production of flavonoids, such as pinocembrin, can be challenging due to a number of factors. One challenge is the limited availability of natural sources, as many flavonoids are found in relatively small quantities in certain plants (Zhou et al., 2014). The chemical synthesis of some flavonoids can also be complex, requiring a number of steps and increasing the cost and difficulty of production (Selepe and Van Heerden, 2013). Additionally, some flavonoids are chemically unstable and can decompose or degrade easily (Lu et al., 2019), making them difficult to produce and store. A promising alternative for the synthesis of flavonoids is through microbial biofactories that can be easily upscaled (Pandey et al., 2016; Okoye et al., 2023). Considerable progress has been made in establishing biosynthetic pathways for several flavonoids in industrially relevant microbial host like *E. coli* (Huang et al., 2022), C. glutamicum (Wu et al., 2022) and yeast (Tartik et al., 2023). In this perspective, we recently engineered enzyme cascades in *E. coli* BL21 (DE3) for the synthesis of different flavonoids (Kufs et al., 2020). By expressing a CoA-Ligase from Nicotiana tabacum and a chalcone synthase from *Arabidopsis thaliana* different flavonoids, including pinocembrin, could be produced by adding derivatives of cinnamic acid as the substrate. However, a major challenge in microbial production of flavonoids is that improvement of production yields is required to make this approach economically viable. For many flavonoids, the synthetic production methods cannot economically compete with extraction from natural sources (Sheng et al., 2020; Okoye et al., 2023; Tous Mohedano et al., 2023). Thus, alternative strategies for improving the availability of such flavonoids for medical use are highly desired.

A possible alternative approach to make the availability of flavonoids affordable could be by engineering probiotic bacteria to produce them directly in the body. However, this strategy would involve the inclusion of genetically engineered bacteria in the body and therefore their biocontainment at the specific application site must be ensured. Furthermore, as bioactive compounds, the quantity and duration of production of the flavonoids need to be controlled.

In this study, the first steps towards realizing such an approach have been taken based on an engineered living material (ELM) design. In ELMs, metabolically active microbes are contained and retained inside a material that allows diffusion of nutrients, oxygen and metabolic products. Due to these capabilities, ELMs are envisioned as living drug delivery devices for the production and delivery of drugs inside the body (Rodrigo-Navarro et al., 2021). By introducing genetic switches (Rivera-Tarazona et al., 2021; Dhakane et al., 2023) or controlling the mechanical and diffusion properties of the embedding matrix (Priks et al., 2020; Bhusari et al., 2022; Bhusari et al., 2023), drug delivery can be controlled. Herein, we present an ELM consisting of probiotic bacteria engineered to produce a flavonoid encapsulated within a poly(vinylalcohol) (PVA) hydrogel film. We first adapted the previously mentioned pinocembrin-producing enzyme cascade (Kufs et al., 2020) developed in *E. coli* BL21(DE3) for constitutive expression in the probiotic *E. coli* Nissle 1917 strain. This bacterium has been extensively engineered as living biotherapeutic for treating different pathologies (Lynch et al., 2022) and has been previously used in ELMs for therapeutic purposes (Praveschotinunt et al., 2019; Chen et al., 2023). We demonstrate that the engineered strain is capable of converting cinnamic acid to pinocembrin in the hours timescale. When encapsulated in the biocompatible PVA hydrogel, the resulting ELM can convert cinnamic acid to pinocembrin without releasing the biofactories. Finally, the possibility to tune output levels by varying cinnamic acid concentrations and sustain production for at least a month using this ELM is shown.

## 2 Materials and methods

### 2.1 Construction of plasmids for constitutive production of pinocembrin

To obtain plasmids expressing the selected genes, we modified the previously reported pMGE-T7 plasmid (Kufs et al., 2020) by replacing the T7-lacO promoter with 3 different constitutive elements (Supplementary Figure S1). The sequences were chosen from the iGem catalogue (https://parts.igem.org). The selected constitutive promoters were BBa_K823004 (here called K2), BBa_J45993 (OY) and BBa_J01006 (R10). We designed three different synthetic DNA elements (sequences are given below), which were amplified using the primers pVV.rec.F and pVV.rec.R and cloned into the basal vector pVV-01 (Hoefgen et al., 2018). Then, the gene encoding for the 4-coumaroyl-CoA ligase (4Cl) and type III polyketide synthase (Chs) were amplified by pMGE-4CL and pMGE-Chs, respectively, using primers pMGEt1 and pMGEt2. This resulted in fragments that could be integrated into the vectors by seamless cloning. Finally, each plasmid containing the 4Cl gene was recombined with the corresponding plasmid harbouring the Chs gene, resulting in an expression vector containing both genes under the control of the selected constitutive promoter. The obtained plasmids and the used oligonucleotides are reported in Tables 1, 2, respectively.

**TABLE 1:** Plasmids used in this study.

Plasmid name	Relevant features	Reference
pVV.01	pUC-ORI, kanR	Hoefgen et al. (2018)
pMGE-T7	pVV.01, T7 promoter, terminators	Kufs et al. (2020)
pMGE_CHS	pMGE-T7, CHS *Arabidopsis thaliana*	Kufs et al. (2020)
pMGE_4CL2	pMGE-T7, 4CL2 *Nicotiana tabacum*	Kufs et al. (2020)
pMGE-K2	pVV.01, K2 promoter, terminators	This study
pMGE-K2-4CL	pMGE-K2, 4CL2	This study
pMGE-K2-Chs	pMGE-K2, CHS	This study
pMGE-K2-nar	pMGE-K2, 4CL2-CHS	This study
pMGE-OY	pVV.01, OY promoter, terminators	This study
pMGE-OY-4CL	pMGE-OY, 4CL2	This study
pMGE-OY-Chs	pMGE-OY, CHS	This study
pMGE-OY-nar	pMGE-OY, 4CL2-CHS	This study
pMGE-R10	pVV.01, R10 promoter, terminators	This study
pMGE-R10-4CL	pMGE-R10, 4CL2	This study
pMGE-R10-Chs	pMGE-R10, CHS	This study
pMGE-R10-nar	pMGE-R10, 4CL2-CHS	This study

**TABLE 2:** Oligonucleotides used in this study.

Primer name	Sequence (5‘-3‘)
pVV.rec.F	CTC​ACG​TTA​AGG​GAT​TTT​GGT​TC
pVV.rec.R	AAT​CGA​ACT​TTT​GCT​GAG​TTG​GG
pMGEt1	AAT​TTG​CGC​AAG​AAG​GAG​ATA​G
pMGEt2	CCC​CGC​GGG​GTT​GCT​TCA​C

### 2.2 Culture of *E. coli* Nissle 1917 and transformation of the plasmids

To prepare chemically competent *E. coli* Nissle 1917 cells, an overnight culture was diluted 1:100 in fresh LB media and grown at 37°C and 200 rpm until an OD600 nm of 0.3–0.4 was reached. The culture was chilled on ice for 30 min and subsequently centrifuged at 3,500 rpm, 4°C and 10 min. The supernatant was discarded and the cell pellet was washed twice with ice-cold 0.1 M CaCl2. The cells were resuspended in 0.1 M CaCl2 with 20% Glycerol and stored on ice or at −80°C until transformation. For transformation, the chemically competent *E. coli* Nissle 1917 cells were mixed with 100 ng of pMGE-K2-nar plasmid DNA and incubated on ice for 30 min. Transformation was performed with a heat-shock at 42°C for 30 s and subsequent cooling on ice for 2 min. For recovery, the cells were resuspended in 950 µL LB medium and incubated at 37°C and 180 rpm for 1 h. After incubation the cells were centrifuged for 2 min at 6,000 rcf and the 900 µL of the supernatant was discarded. The cell pellet was resuspended in the remaining supernatant, which was then spread on an LB agar plate containing 50 μg/mL kanamycin for selective growth of plasmid containing bacteria and incubated overnight at 37°C.

### 2.3 Characterization of pinocembrin production levels

For studying the pinocembrin production response to initial concentration of cinnamic acid, a 50 mL over-night culture of *E. coli* Nissle K2 in LB medium containing 50 μg/mL Kanamycin (LB Kn50) was prepared from a cryo-stock and incubated at 23°C and 180 rpm to prevent overgrowth. The temperature was subsequently set at 37°C in the morning and exponentially grown cells (OD600 nm of 1) were harvested. These cells were used to inoculate 3 mL of LB Kn50 containing 0.016–4 mM trans-cinnamic acid (serially diluted by a factor of 2 starting from 4 mM) at an OD600 nm of 0.1 in 12 mL cultivation tubes. 1 mL samples were taken after 24 h and spun down with a table centrifuge at 6,500 rcf for 3 min 750 μL supernatant were transferred to a fresh reaction tube and stored at −20°C for LC/MS measurement. The cultures were incubated at 37°C and 250 rpm.

For observing Pinocembrin synthesis over time an overnight culture was prepared as described above. Exponentially grown cells from were used to inoculate 50 mL LB Kn50 + 4 mM trans-cinnamic acid at an OD600 nm of 0.1 in a 500 mL baffled conical flask. The culture was incubated at 37°C and 250 rpm. 1 mL samples were taken every 2 h until 8 h of incubation after which 1 mL samples were taken at 24 h and 48 h. Cells in these samples were spun down in a table centrifuge at 6,000 rcf for 3 min 750 μL supernatant was transferred to a fresh reaction tube and stored at −20°C for LC/MS measurement.

### 2.4 Encapsulation of the bacteria in PVA gels

Poly(vinyl alcohol) (Mowiol 18-88, 130,000 Da, 86.7%–88.7% hydrolysis degree, Sigma Aldrich) was functionalized with vinylsulfone (VS) reactive groups to obtain PVA-VS following a reported protocol (Puertas-Bartolomé et al., 2023). A 10 %w/v PVA-VS solution in water was mixed with the bacteria suspension in LB Kn50 and Lithium phenyl-2,4,6-trimethylbenzoylphosphinate photoinitiator (LAP, Sigma Aldrich) solution in 2x LB Kn50 to obtain a final PVA-VS concentration of 5% w/v, a 0.5% w/v concentration of LAP and a bacterial OD600 nm of 0.05 (∼4 × 106 cells/mL) in the mixture. Hydrogels were prepared by photoinitiated radical crosslinking of the VS groups using a UV light source (365–480 nm) at irradiance of 6 mW/cm2 for 2 min.

For microscopy studies, 10 µL of the mixture were photocroslsinked inside an ibidi µ-Slide angiogenesis micro-well plate. To characterize flavonoid production, ELMs in the form of bacterial hydrogel films supported on glass coverslips (13 mm diameter, 16 mm thickness) were prepared. The glass was previously coated with 3-(trimethoxy silyl) propyl acrylate to facilitate covalent attachment of the hydrogel to the glass surface. The films contained two layers and were fabricated in two steps. First, 28 µL of the mixture were deposited in the center of a 6 mm diameter polydimethylsiloxane (PDMS) mould and pressed against the coated coverslip. Photocrosslinking (365–480 nm at irradiance of 6 mW/cm2 for 2 min) and removal of the mould rendered a film of the hydrogel attached to the glass slide. Next, a second PDMS ring (10 mm inner diameter) was placed concentrically on top of the thin PVA film. 80 μL of 10 %w/v PVA-VS aqueous solution containing 0.5% w/v of LAP initiator were deposited in the ring to fully cover the bacteria hydrogel with an enveloping layer that did not contain bacteria. After photocrosslinking (365–480 nm at irradiance of 6 mW/cm2 for 2 min) and removal of the mould, bilayer hydrogel films with 10 mm final diameter and 1 mm thickness were obtained.

### 2.5 Microscopy of PVA encapsulated bacteria

Bacteria hydrogel samples from days 0, 2, 4, 7, and 14 in the ibidi µ-Slide angiogenesis micro-wells were imaged using a Zeiss LSM 880 confocal laser scanning microscope (Zeiss, Oberkochen, Germany). Images were captured using a Zeiss Plan-Apochromat 63x/1.4 Oil DIC M27 objective with detection wavelengths 482–544 nm and 584–718 nm, and laser wavelengths of 488 and 543 nm respectively for live and dead bacterial populations. Z-stacks of 18.44 µm were taken in a z-step size of 0.45 µm. Images of a size of 134.95 × 134.95 µm were acquired (1,024 × 1,024 pixels), two-fold line averaging, and pixel dwell time of 0.42 µs.

Representative images were obtained using the maximum intensity Z-projection (Zen 2.3 SP1, Zeiss, Oberkochen, Germany). The Imaris surface tool (Imaris v9.8, Bitplane, Zurich, Switzerland) was used to calculate the 3D volume of live and dead bacteria. The surfaces were generated with the smooth function set to 0.264 µm, the diameter of largest Sphere to 10 μm, and the automatic threshold. The volume fraction of live/dead bacterial colonies in the hydrogel samples was calculated as the sum of all live/dead colony volumes divided by the total volume of the imaged region (335,820 µm^3^). The Live % was calculated as the volume of live colonies divided by the total volume of the live and dead colonies.

### 2.6 Characterization of pinocembrin production from the gels

For quantitation of pinocembrin production, the bilayer ELM films were incubated with four different concentrations of cinnamic acid. Incubation was performed in a 24-well plate first at 37°C with 400 µL of LB Kn50 media for 3 days to allow bacteria to grow. From day 4 on, the bacteria hydrogels were covered with 400 µL of LB Kn50 + 4, 2, 0.5 and 0.125 mM cinnamic acid media. Five technical replicates were prepared for each condition, while 1 replicate was incubated in LB Kn50 as control. The supernatant was taken every 24 h and replaced with corresponding fresh media. 100 μL samples from the supernatants were centrifuged in a table centrifuge, transferred to a fresh 1.5 mL Eppendorf reaction tube, and stored at −20°C until LC-MS measurement. For quantitation of sustained pinocembrin production, the ELM films were first incubated at 37°C in a 24-well plate with 400 µL of LB Kn50 media for 3 days to allow bacteria to grow. From day 4 on, the films were covered with 400 µL of LB Kn50 + 4 mM cinnamic acid media and LB Kn50 as control and kept at 37°C for 3 days during which the medium was exchanged each day. Six technical replicates were prepared for both conditions. The supernatant was taken every 24 h and replaced with corresponding fresh media. 100 μL samples from the supernatants were centrifuged in a table centrifuge, transferred to a fresh 1.5 mL Eppendorf reaction tube, and stored at −20°C until LC-MS measurement.

### 2.7 LC/ESI QTOF-MS method development for quantification of pinocembrin in supernatants

LC/ESI QTOF-MS analysis is performed on a 1,260 Infinity LC in combination with a 6545A high-resolution time-of-flight mass analyzer, both from Agilent Technologies (Santa Barbara, CA, United States). Separation of 1 µL of sample is performed using a Poroshell HPH-C18 column (3.0 mm × 50 mm, 2.7 µm) equipped with the same guard column (3.0 mm × 5 mm, 2.7 µm) by a linear gradient from (A) ACN +0.1% formic acid to (B) water +0.1% formic acid at a flow rate of 500 μL/min and a column temperature of 45°C. Gradient conditions are as follows: 0–0.5 min, 50% B; 0.5–2 min, 50%–37% B; 2–4 min 37%–50% B, 4–5 min 50% B at 1,500 μL/min (column cleaning), 5–7 min 50% B down to 500 μL/min. After separation, the LC flow enters the dual AJS ESI source set to 4,000 V as capillary voltage, 50 psi nebulizer gas pressure and 12 L/min dry gas flow, and 350°C dry gas temperature. The TOF parameters used are extended dynamic range (2 GHz), 160 V fragmentor and 45 V skimmer voltage. The mass spectra are acquired in the time interval of 0.75–2 min in full scan mode in the range m/z 100-1,000 with a spectra rate of 1/s. To determine the formed Pinocembrin, the negative charged mass [M-H]- at m/z 255.0667 Da were extracted and automatically integrated using Mass Hunter software. Standards were prepared from Pinocembrin stock solution of 31 μg/mL in EtOH, further dilutions were done in mobile phase (50%A, 50%B) or media. Samples of cinnamic acid screening were diluted 1:100 with mobile phase (50% H2O and 0.1% formic acid +50% Acetonitrile and 0.1% formic acid) in a total volume of 1 mL. Samples for Pinocembrin synthesis over time were diluted 1:10 for samples of 0–8 h in a total volume of 100 µL and 1:100 in a total volume of 1 mL.

### 2.8 Details regarding statistical analysis

All data presented were obtained from experiments that included replicates. The information regarding the type and number of replicates are included in the captions of the figures where the data has been shown. Similarly any statistical analysis performed has also been described in the captions. The graphs were made with Origin Pro 2023 software, which was also used for statistical analysis.

## 3 Results

**FIGURE 1:**
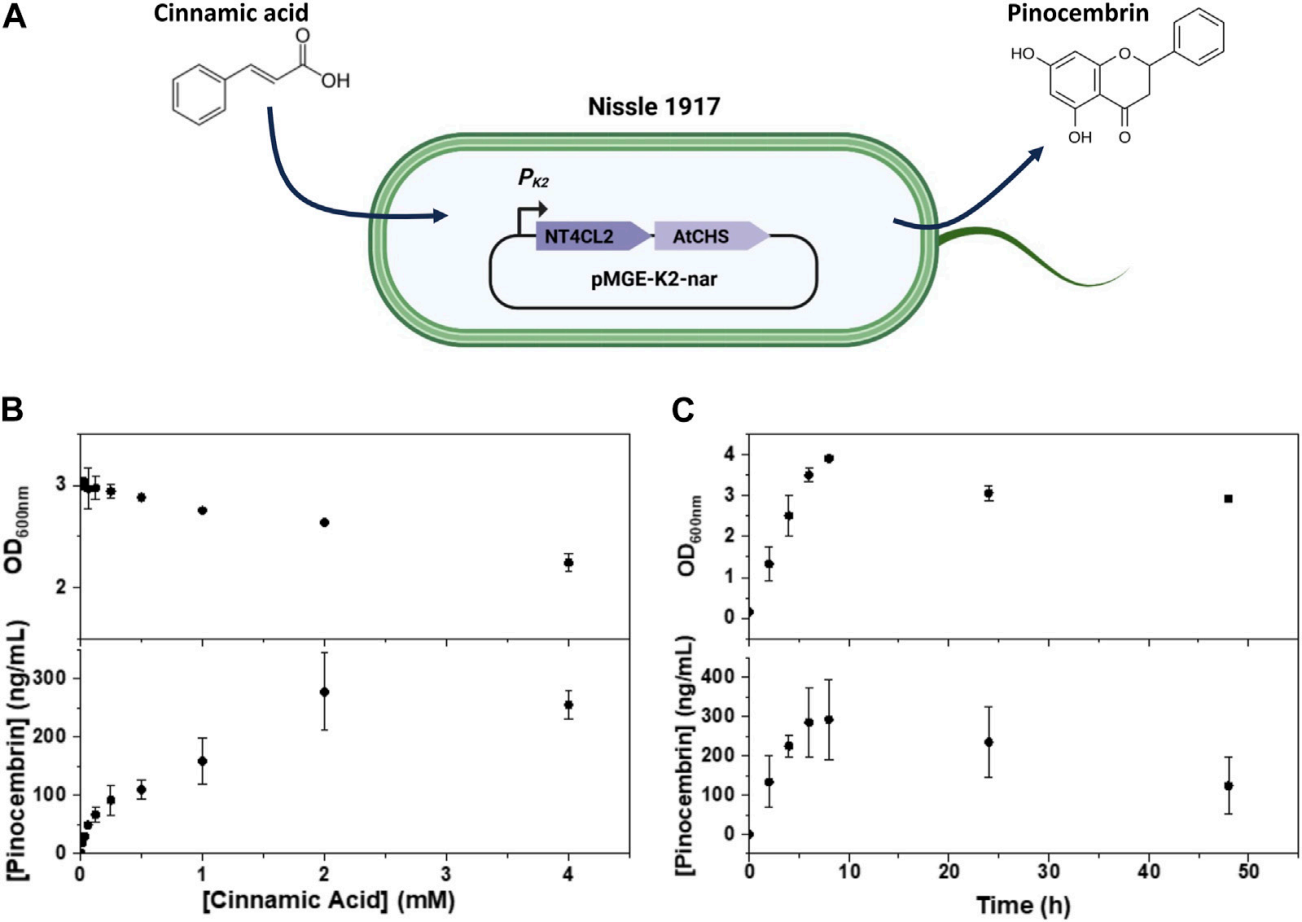
**(A)** Scheme representing the pMGE-K2-nar plasmid encoded in *E. coli* Nissle 1917 with the PK2 promoter driving expression of the enzymes converting cinnamic acid to pinocembrin. **(B,C)** Analysis of bacterial growth and pinocembrin production in cultures of *E. coli* Nissle 1917 pMGE-K2-nar in the presence of cinnamic acid at different concentrations **(B)** and at different time-points **(C)**. Symbols are means and whiskers are standard deviation from 2 **(B)** and 3 **(C)** independent experiments.

In a previous study, pinocembrin biosynthesis in an *E. coli* lab strain was achieved by expressing genes encoding a CoA-ligase from Nicotiana tabacum (NT4CL2) and a chalcone synthase from *A. thaliana* (AtCHS) under control of an IPTG-inducible T7-lacO promoter (Kufs et al., 2020). Towards applicability in the body, it was desirable to encode these enzymes in a biocompatible strain and remove the need for induction. Accordingly, in this study we used *E. coli* Nissle 1917 (Figure 1A), which is a commercial probiotic (Mutaflor) extensively explored as a chassis for drug delivery in the body (Chen et al., 2023). To eliminate the need for induction, we replaced the T7-lacO promoter with 3 different constitutive promoters and tested their capacity to convert cinnamic acid to pinocembrin. The promoters, PK2 (BBa_K823004) and PR10 (BBa_J01006) are expected to be more active in the exponential growth phases, while POY (BBa_J45993) is more active in the stationary phase. When plasmids encoded with the enzymes driven by each promoter were transformed in this strain, pinocembrin production in cultures containing cinnamic acid was only observed with the strain bearing the PK2 promoter (Supplementary Figure S2). Notably, the inserts could not be detected in sequencing results when plasmids containing the POY and PR10 promoters were transformed in *E. coli* Nissle 1917, suggesting possible incompatibility of these constructs with the strain.

The effects of exposing *E. coli* Nissle 1917 encoded with the pMGE-K2-nar plasmid to increasing concentrations of cinnamic acid (0.016–4 mM) were tested. First, we checked if cinnamic acid affected bacterial growth rate in liquid culture by following the increase in cell density (OD600 nm) over 24 h. Cell densities reached steady OD600 nm values of 2–3 for cinnamic acid concentrations up to 4 mM although the OD600 nm values dropped with increasing cinnamic acid concentrations (Figure 1B). This suggested that *E. coli* Nissle 1917 growth was affected by increasing concentrations of cinnamic acid. Next, pinocembrin levels in medium were quantified. A cinnamic acid concentration dependent increase in pinocembrin levels from 15 to 300 ng/mL was observed with a peak at 2 mM cinnamic acid. On normalization of the pinocembrin concentrations with final OD600 nm values, the highest mean production rate was with 4 mM cinnamic acid (113 ± 15 ng/mL/OD pinocembrin), although it was not significantly different from the rate at 2 mM cinnamic acid (105 ± 25 ng/mL/OD pinocembrin) (Supplementary Figure S3A). Thus, with 4 mM cinnamic acid, time-resolved conversion behavior at this concentration was tested in liquid cultures for 48 h (Figure 1C). The highest cell density was reached after 8 h during which pinocembrin reached a peak mean concentration of 280 ng/mL. Prolonged incubation for 24 h and 48 h resulted in a decrease of both cell density and pinocembrin concentration. A decrease in cell density is attributed to death of the bacteria in the saturation growth phase. The decrease in pinocembrin concentrations was due to its degradation over time, which we observed in experiments where pure pinocembrin was incubated in a control *E. coli* Nissle 1917 culture (unmodified) and in LB medium for 48 h (Supplementary Figure S3).

**FIGURE 2:**
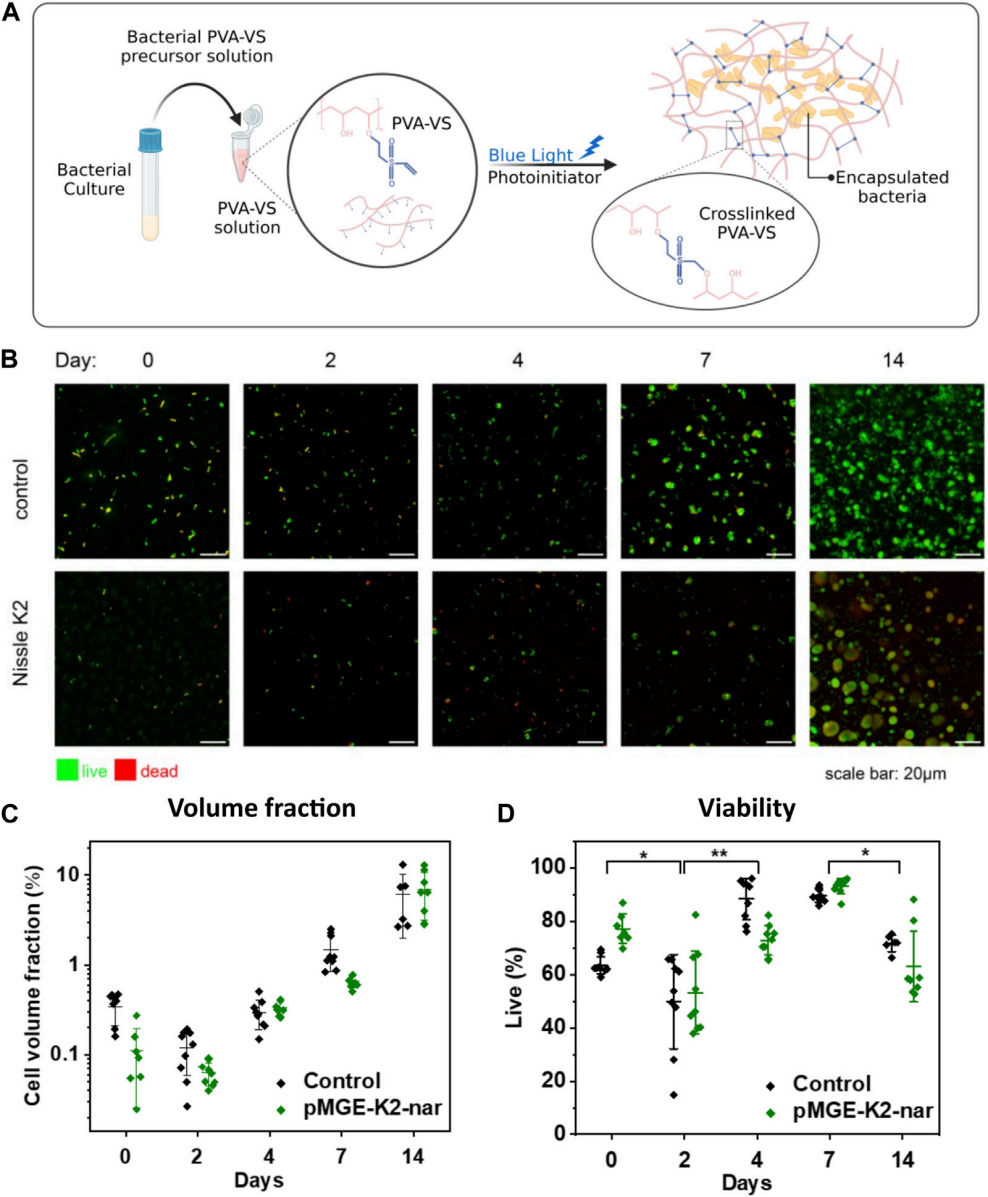
**(A)** Graphical scheme of bacterial encapsulation in a PVA-VS hydrogel matrix that is chemically cross-linked. **(B)** Fluorescence confocal microscopy images of live/dead stained bacteria in PVA-based chemically cross-linked hydrogels grown for up to 14 days. **(C)** Analysis of the volume fraction of the bacteria populations within the imaged volumes over 14 days. **(D)** Analysis of cell viability over 14 days. Each symbol represents values from a single image. Central lines are means and whiskers are standard deviations of values obtained from 3 images at different locations in 3 independent samples. Significance was determined by one-way ANOVA with means comparison by the Tukey test (*p < 0.05, **p < 0.005)

Encouraged by these results, we proceeded to encapsulate the bacteria for the preparation of ELMs using PVA as the polymer matrix. PVA is a synthetic, water-soluble, and biocompatible polymer widely used for the encapsulation of probiotics (Çanga and Dudak, 2021; Corona-Escalera et al., 2022). In this work, the PVA backbone was modified with vinyl sulfone groups to create a stable hydrogel network by chemical crosslinking (Figure 2A). The suitability of this material for the preparation of ELMs supporting bacterial viability and functionality has been recently demonstrated (Puertas-Bartolomé et al., 2023). Bacterial hydrogels containing *E. coli* Nissle 1917 encapsulated in PVA-VS were prepared in well plates and photo-crosslinked. We first characterized the growth and viability of the bacteria within the PVA hydrogels for 14 days using confocal microscopy. Hydrogel films containing control Nissle 1917 (wildtype) or Nissle 1917 bearing the pMGE-K2-nar plasmid were prepared. Both strains grew from single cells on day 0 to rounded colonies from day 7 that continued to increase in size over 14 days (Figure 2B). The volume occupied by the bacteria within the hydrogel reached 7% by day 14 (Figure 2C). Analysis of cell viability using a Live/Dead staining, revealed a drop in bacteria viability on day 2, but a recovery in following days to reach 80% at 7 days and above 50% at 14 days (Figure 2D). Colony volume and viability values were comparable for the control and pMGE-K2-nar strains, revealing that they remained unaffected by expression of the pinocembrin-synthesis enzyme and the presence of 4 mM cinnamic acid in the medium. In conclusion, *E. coli* Nissle strains presented high viability after encapsulation and were able to proliferate within the hydrogels for at least 2 weeks by the formation of spatially contained colonies.

**FIGURE 3:**
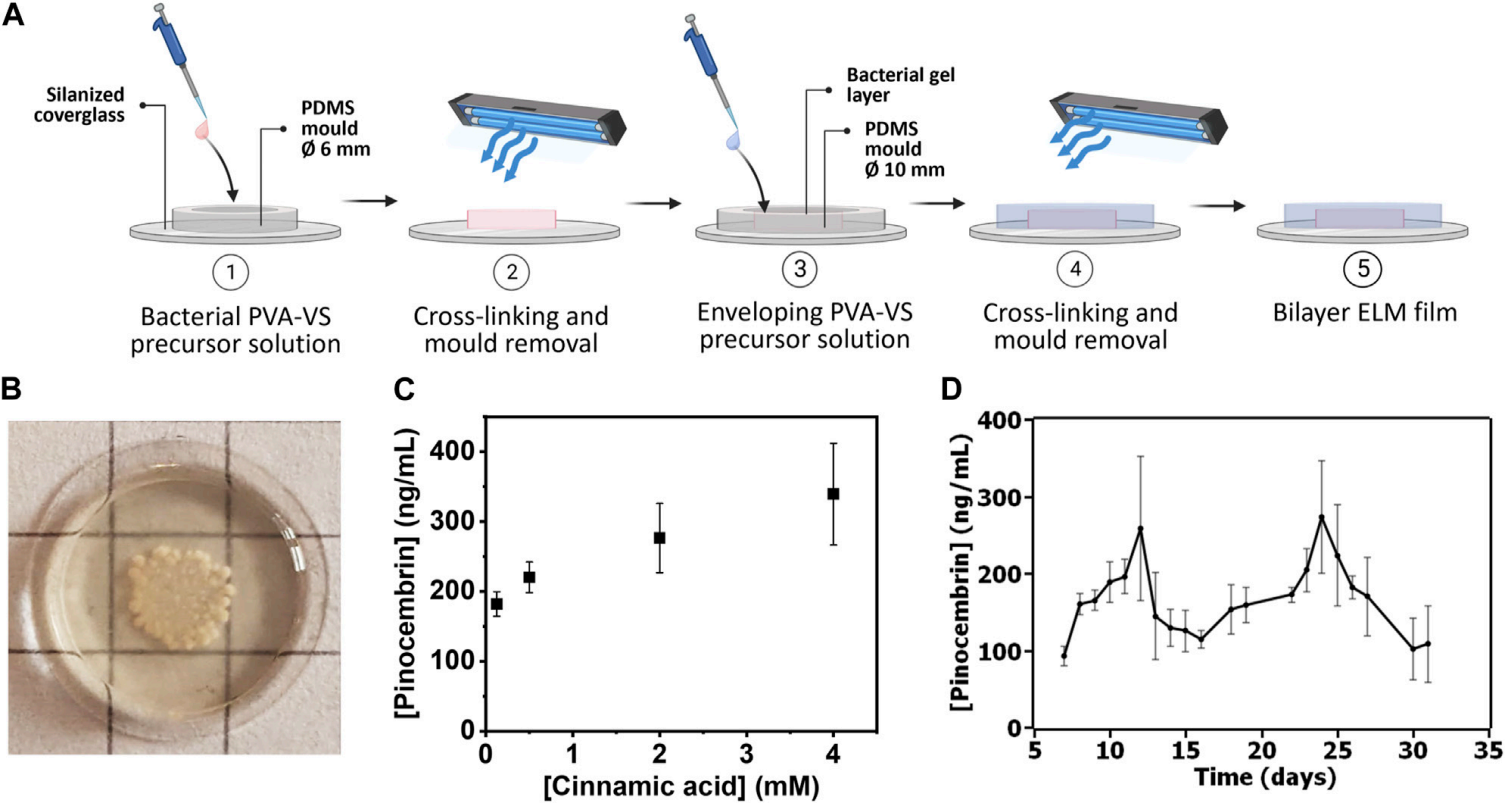
**(A)** Bacterial PVA-VS bilayer ELM films fabrication protocol. **(B)** Photo of a bilayer ELM film with bacterial growth over 13 days. Size of grid sizes = 0.5 cm. More images in Supplementary Figure S5 **(C)** Analysis of pinocembrin production at different cinnamic acid concentrations from the bilayer ELM films. Symbols are means and whiskers are standard deviations from 4 independent bilayer ELM films for each concentration. **(D)** Analysis of long-term production of pinocembrin production from day 7–31 after fabrication of the bilayer ELM films. Symbols are means and whiskers are standard deviations from 3 independent bilayer ELM films.

In the single layer films, we observed outgrowth and leaking of bacteria from the hydrogel as observed by microscopy of the surrounding medium (data not shown). To prevent this, bilayer films were fabricated wherein the bacterial hydrogel was enveloped by a bacteria-free hydrogel layer (Dhakane et al., 2023). The films were supported on a glass cover slip, to which the gel was covalently bonded (Figures 3A, B). Bacterial leakage/outgrowth from the bilayer films was tested during 31 days by analyzing the supernatant on nutrient agar plates. Among 6 samples, one sample leaked on day 9, another sample on day 14, two more on day 24 and two samples did not leak after 31 days (Supplementary Figure S4). We did not observe degradation of the hydrogels in the samples that leaked bacteria when checked by eye (Supplementary Figure S5) or under the microscope (Supplementary Figure S6). This result, taken together with the fact that leakage occurred at very different timepoints among the different samples, led us to think that leakage is due to imperfections in the manual fabrication of the films. One possibility is contamination of the enveloping layer with a few bacterial cells that were then able to grow out when they formed large colonies (Supplementary Figure S6). Another potential point of failure could be the interface between the hydrogel and the glass layer, since we observed peeling off the hydrogel in some of the samples (Supplementary Figure S6). Nevertheless, the long-term bacterial leakage experiment evidenced that the bilayer films could contain the bacteria for a long time if the variability in the manual process can be avoided, possibly through automation.

Based on the microscopy results which showed colonies on day 6, this time scale was selected for the quantification of pinocembrin production from cinnamic acid added to the medium. By increasing the concentration of cinnamic acid from 0.125 to 4 mM, pinocembrin production on day 6 increased from 200 to 320 ng/mL (Figure 3C). This indicates the possibility to tune the output with different input concentrations. We assessed for how long conversion could be sustained with daily renewal of medium at 4 mM cinnamic acid concentration. The average pinocembrin release over this period varied from 100 to 250 ng/mL from day 7–31 (Figure 3D). These results indicate that long-term conversion of pinocembrin from cinnamic acid could be realized with an ELM approach. The fluctuation in the pinocembrin levels over this period of time suggests possible variations in the viability or functionality of the encapsulated bacterial populations, in line with the 14-day viability results in Figure 2C. Interestingly, conversion efficiency was similar to what was observed in liquid cultures, suggesting that diffusion of cinnamic acid into and pinocembrin out of the hydrogels was not a major limiting factor and that the bacteria in the encapsulated form retain their properties.

## 4 Discussion and conclusion

In this study, we report an ELM capable of catalytically producing a high-value bioactive compound by conversion of low-cost food grade cinnamic acid. With bilayer ELM films containing probiotic bacteria encoded with 2 enzymes, we demonstrated that conversion of cinnamic acid to pinocembrin could be tuned at a single time-point based on the precursor concentration. Production was sustained for over 3 weeks. The bilayer ELM films rendered conversion levels comparable to liquid cultures, indicating that diffusion of the substrate is not a major limiting factor and does not impact the reaction rate. Our previous studies have shown that other bioactive products could be produced from the same 2 enzymes by varying the input precursor (Kufs et al., 2020). For instance, coumaric acid was converted to naringenin, caffeic acid to eriodictyol, ferulic acid to homoeriodictyol and various cinnamic acid analogues to corresponding pinocembrin analogues. Thus, our ELM design could potentially be extended to be used to selectively produce a number of bioactive compounds depending on the precursors supplied to it.

A limitation of this study is the low conversion efficiency of cinnamic acid into pinocembrin. With the current non-optimized enzyme cascade, the amount of pinocembrin produced (100—400 ng/mL) was 3 orders of magnitude lower than that of the precursor (20—600 μg/mL). In terms of costs, cinnamic acid (>95% purity) can be purchased at retail prices as low as 0.1 EUR/g, while pinocembrin (>95% purity) is over 4 orders of magnitude more expensive around 7000 EUR/g (sources—Sigma Aldrich). Thus, the conversion efficiency in the current system could provide a cost advantage if it can be maintained in the body. To realize health-promoting effects of pinocembrin, higher concentrations by 10- to 100-fold have been reported based on *in vitro* cell culture and *in vivo* animal studies (Soromou et al., 2013; Gong, 2021). Thus, an improvement in conversion efficiencies might be still required. One possibility for an 18-fold boost of pinocembrin production in *E. coli* up to 40 μg/mL was reported by regulating cinnamic acid metabolism (Cao et al., 2016). In another report, pinocembrin yields could be increased to nearly 200 μg/mL by modifying the antibiotic resistance cassette to kanR from ampR and using a CoA ligase from soybeans (Dustan et al., 2020). Such optimization could improve the precursor conversion efficiency and rate of production of the bioactive compound from the ELM. Nevertheless, the low production yields currently achieved could still provide the possibility to use these ELMs provide highly localized micro-dosed effects in the body, like anti-inflammatory and antioxidant effects. Further in-depth studies with suitable model systems to test such effects will be explored in future studies.

For application inside the body, ELMS with formats compatible with the tissue type are needed PVA hydrogels can be fabricated in various formats, like microcapsules (Han and Hong, 2006), electrospun meshes (Akbar et al., 2018) or 3D printed scaffolds (Meng et al., 2020). These diverse possibilities highlight the potential for catalytic ELMs to be developed as living medical devices capable of promoting health at low-cost.

## Data Availability

The raw data supporting the conclusion of this article will be made available by the authors, without undue reservation.
