# Supplementary material for: Engineered living materials for the conversion of a low-cost food-grade precursor to a high-value flavonoid
Source: Front Bioeng Biotechnol. 2023 Nov 28;11:1278062. doi: 10.3389/fbioe.2023.1278062 (PMC10715425; doi:10.3389/fbioe.2023.1278062)
Supplement: Supplementary file 1 [file DataSheet1.DOCX]

***Supporting information***

**Engineered living materials for the conversion of a low-cost food-grade precursor to a high-value flavonoid**

**Florian Riedel,^1^ Maria Puertas Bartolomé,^1,5^ Lara Luana Teruel Enrico,^1,2^ Claudia Fink-Straube,^1^ Cao Nguyen Duong,^1^ Fabio Gherlone,^3,4^ Ying Huang,^3,4^ Vito Valiante,^3,4^ Aránzazu del Campo,^1,2^ Shrikrishnan Sankaran^1*^**

^1^INM - Leibniz Institute for New Materials, Saarbrücken, Germany

^2^Chemistry Department, Saarland University, 66123 Saarbrücken, Germany

^3^Biobricks of Microbial Natural Product Syntheses, Leibniz Institute for Natural Product Research and Infections Biology - Hans Knöll Institute, Jena, Germany

^4^Faculty of Biological Sciences, Friedrich Schiller University Jena, Jena, Germany

^5^Current affiliation – Bioforge Lab (Group for Advanced Materials and Nanobiotechnology), CIBER-BBN, Edificio LUCIA, Universidad de Valladolid, Spain

***Correspondence:**

Dr. Shrikrishnan Sankaran

Email: [Shrikrishnan.sankaran@leibniz-inm.de](mailto:Shrikrishnan.sankaran@leibniz-inm.de)

**Synthetic DNA fragments used for subcloning**

Promoter regions are shown in capital letters, while terminators are underlined.

>K2 (based on BBa_K823004)

ctcacgttaagggattttggttctagagatgttt**AAACTTGACGGCTAGCTCAGTCCTAGGTACAGTGCTAGCAATT**tgcgcaagaaggagatagatatctgcactgagtgaagcaaccccgcggggcctcttcgggggtctcgcggggttttttgctgaaagcaaataaaacgaaaggctcagtcgaaagactgggcctttcgttttatcctcgcgtaaccccttggggcctctaaacgggtcttgaggggttttttg**AAACTTGACGGCTAGCTCAGTCCTAGGTACAGTGCTAGCAAtt**taaattccttaattaaatcggatcccaactcagcaaaagttcgatt

>OY (based on BBa_J45993)

ctcacgttaagggattttggttctagagatgttt**AAACTGACATCCCGAGCGGTTTCAAAATTGTGATCTATATTTAACAATT**tgcgcaagaaggagatagatatctgcactgagtgaagcaaccccgcggggcctcttcgggggtctcgcggggttttttgctgaaagcaaataaaacgaaaggctcagtcgaaagactgggcctttcgttttatcctcgcgtaaccccttggggcctctaaacgggtcttgaggggttttttg**AAACTGACATCCCGAGCGGTTTCAAAATTGTGATCTATATTTAACAATT**taaattccttaattaaatcggatcccaactcagcaaaagttcgatt

>R10 (based on BBa_J01006)

ctcacgttaagggattttggttctagagatgttt**AAACTTTTCAGGCCGGAATAACTCCCTATAATGCGCCAAATt**tgcgcaagaaggagatagatatctgcactgagtgaagcaaccccgcggggcctcttcgggggtctcgcggggttttttgctgaaagcaaataaaacgaaaggctcagtcgaaagactgggcctttcgttttatcctcgcgtaaccccttggggcctctaaacgggtcttgaggggttttttg**AAACTTTTCAGGCCGGAATAACTCCCTATAATGCGCCAAATT**taaattccttaattaaatcggatcccaactcagcaaaagttcgatt

 
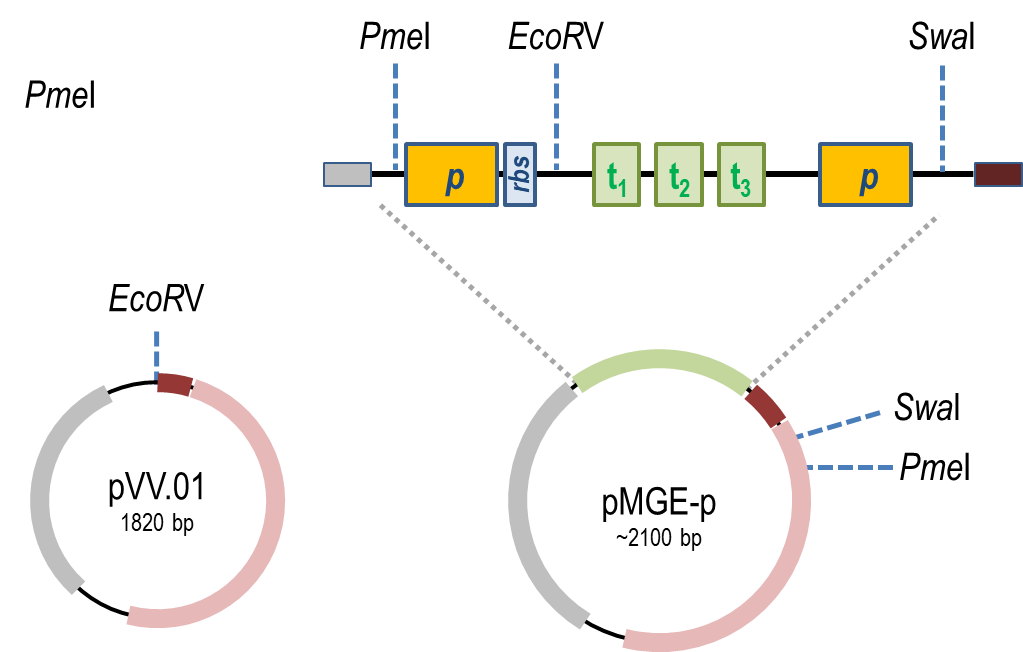


**Figure S1: pMGE-p plasmid series features.** The DNA fragment containing the promoter of interest, an Eco*RV* restriction site and the three terminators, were synthetically produced and inserted in the pVV.01 plasmid upon PCR amplification. The kanamycin resistance cassette is reported in pink while the pUC *ORI* sequence in gray. Figure has been modified from Kufs et al. 2020. *p*: promoter; *rbs*: ribosomal binding site; t: terminator.


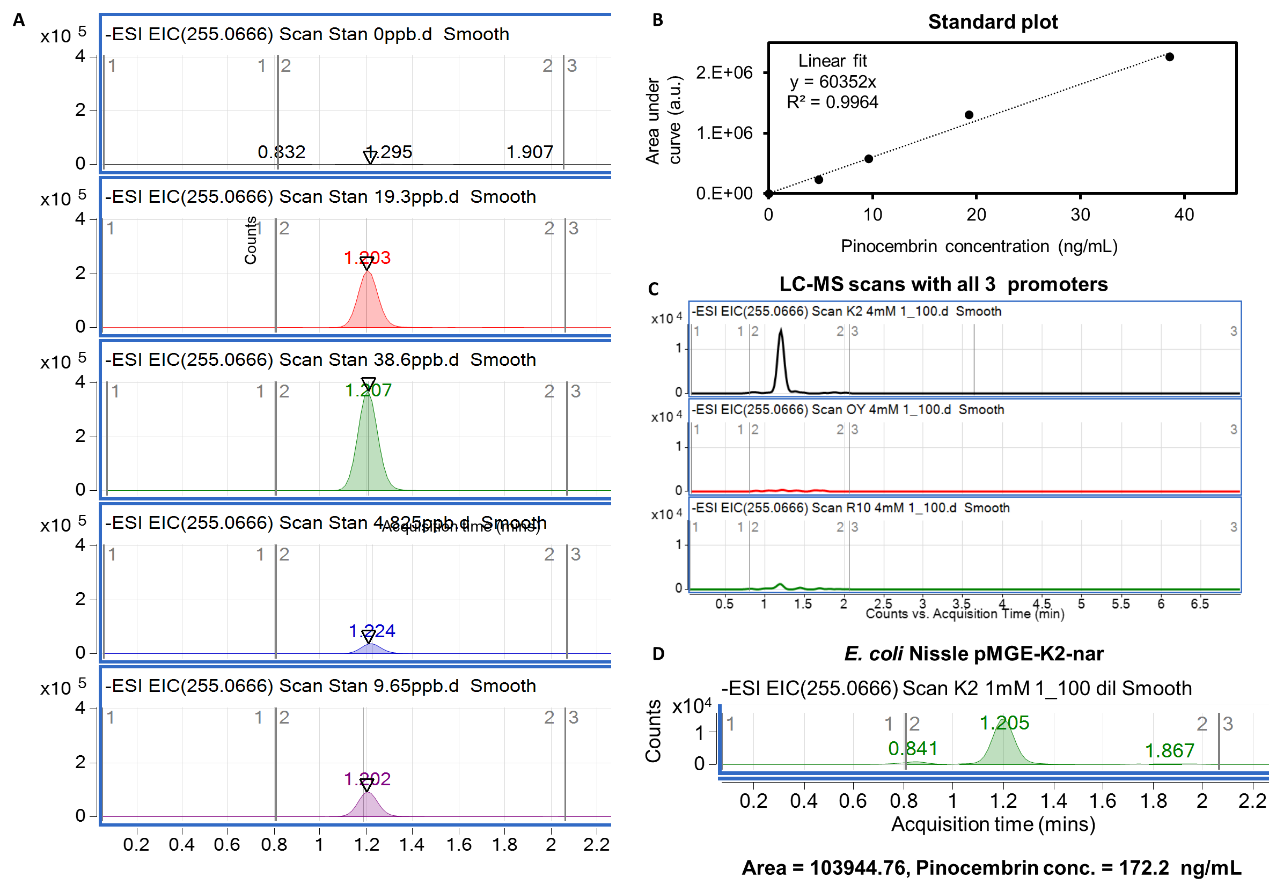


**Figure S2.** A. LC-MS plots with known concentrations of purified pinocembrin in medium for establishing the standard plot to determine pinocembrin concentrations secreted by the engineered bacteria. B. Standard plot of Area under the curve vs Pinocembrin concentrations along with linear fit. C. LC-MS plots of pinocembrin measured in 100-fold diluted cell-free supernatants of 24 h cultures of *E. coli* Nissle 1917 containing pMGE-K2-nar, pMGE-OY-nar, and pMGE-R10-nar plasmids and 1 mM cinnamic acid added at the start of the culture. D. Close-up view of the LC-MS peak associated with the *E. coli* Nissle 1917 pMGE-K2-nar strain with the area used for calculating pinocembrin concentration shaded in green.


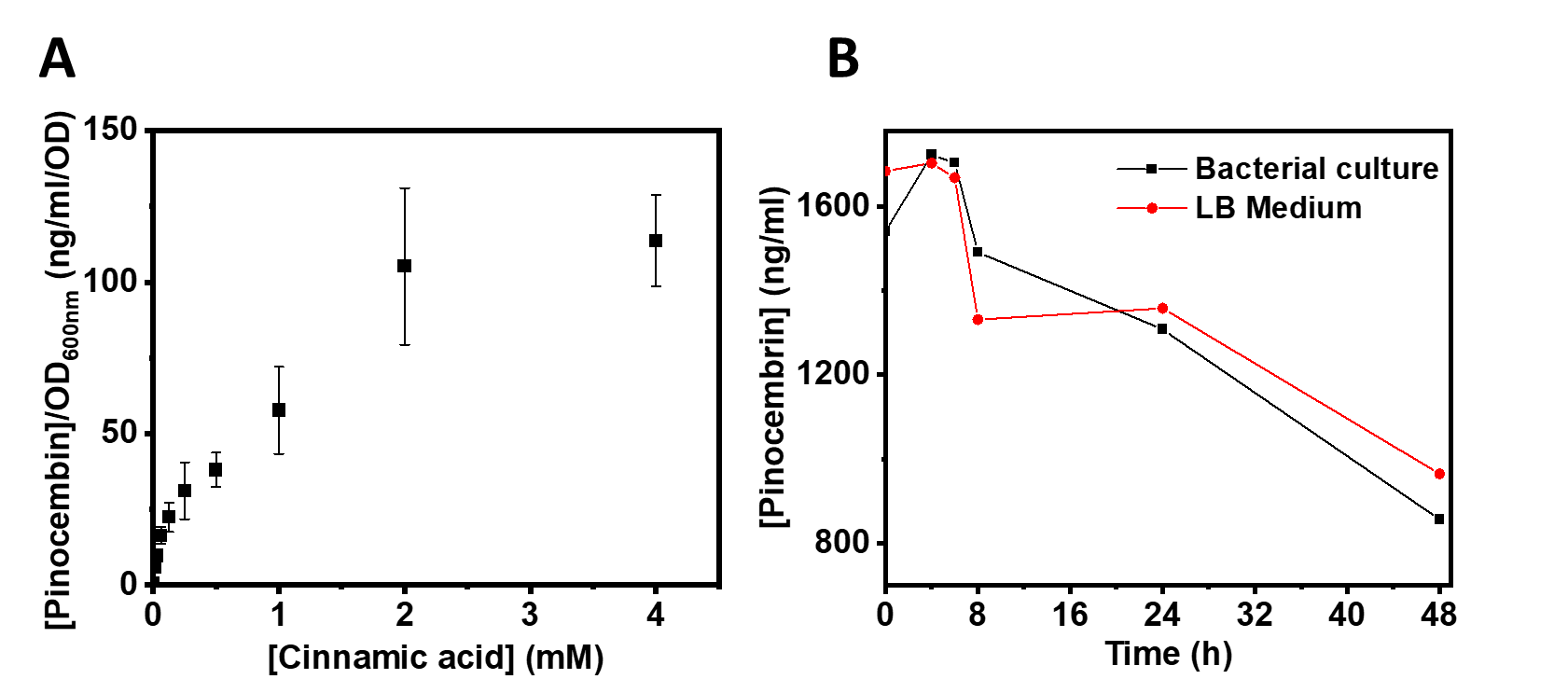


**Figure S3. A.** OD_600nm_-normalized pinocembrin concentrations measured with different initial concentrations of cinnamic acid. **B.** Analysis of pinocembrin degradation over 48 h in control Nissle 1917 bacterial culture and LB medium.


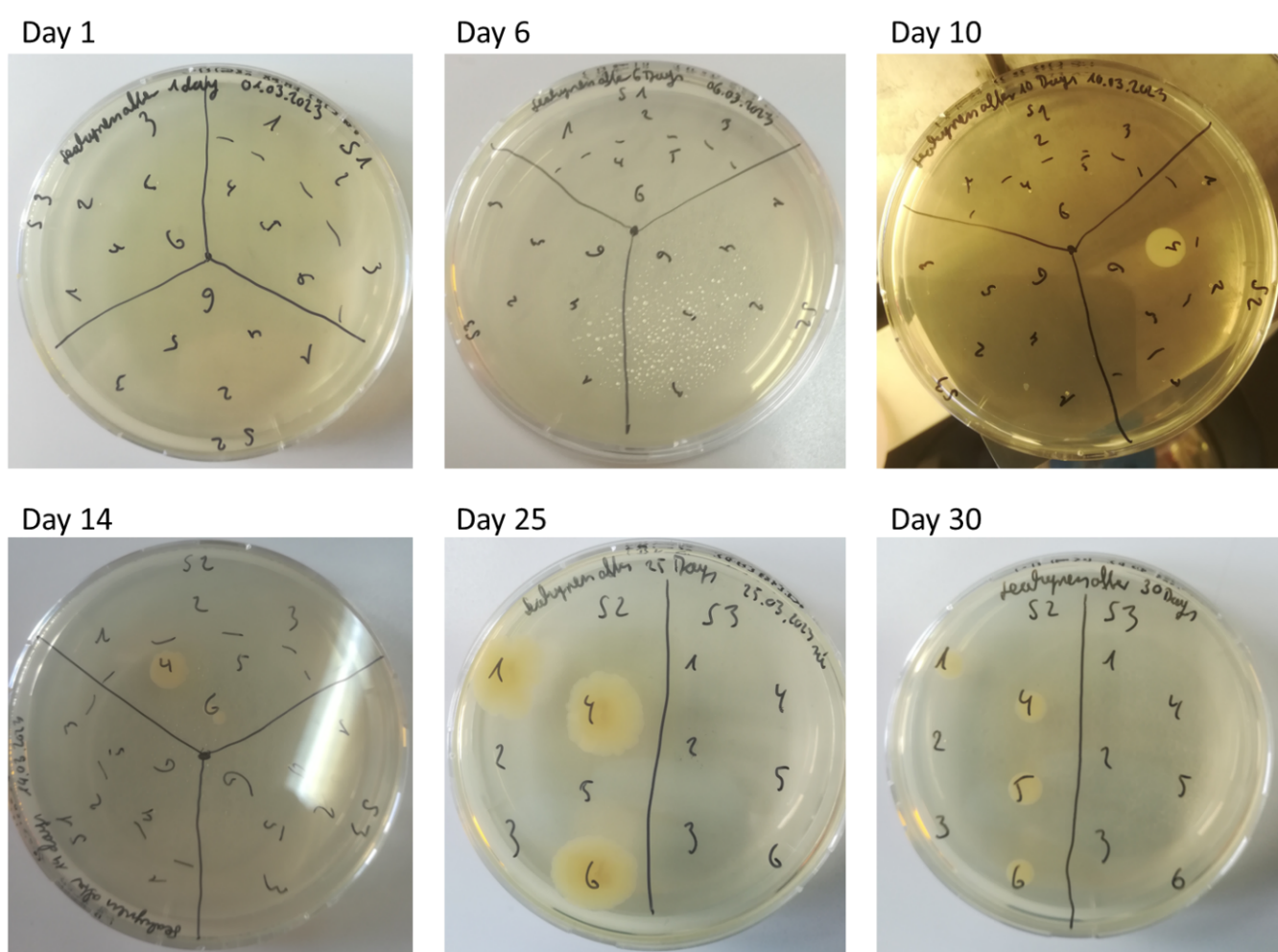


**Figure S4.** Agar plating assay to verify leakiness of ELM films. S1 = Control ELM films without bacteria, S2 = ELM films with E. coli Nissle 1917 pMGE-K2-nar where 4 mM cinnamic acid was added from day 6, S3 = ELM films with E. coli Nissle 1917 pMGE-K2-nar where 4 mM cinnamic acid was added from day 0 due to which bacteria didn’t grow within the films. Supernatants were taken each day from the media in which the ELMs were incubated and spotted on LB-agar plates where the numbers are indicated. Bacterial leakage and contamination of the medium is identified as growth of a lawn-like colony at that spot.


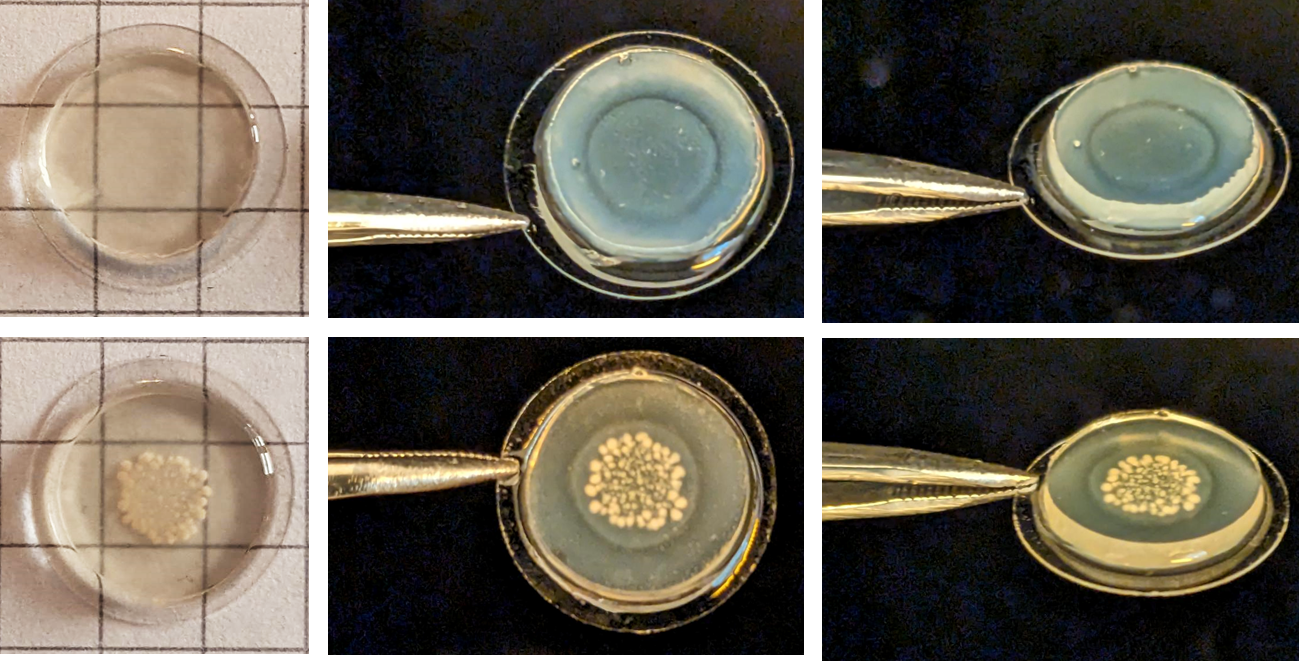


**Figure S5.** Photos of bilayer bacterial-PVA hydrogel ELM films without and with growth of bacterial colonies in the core. Grid square side length = 0.5 cm


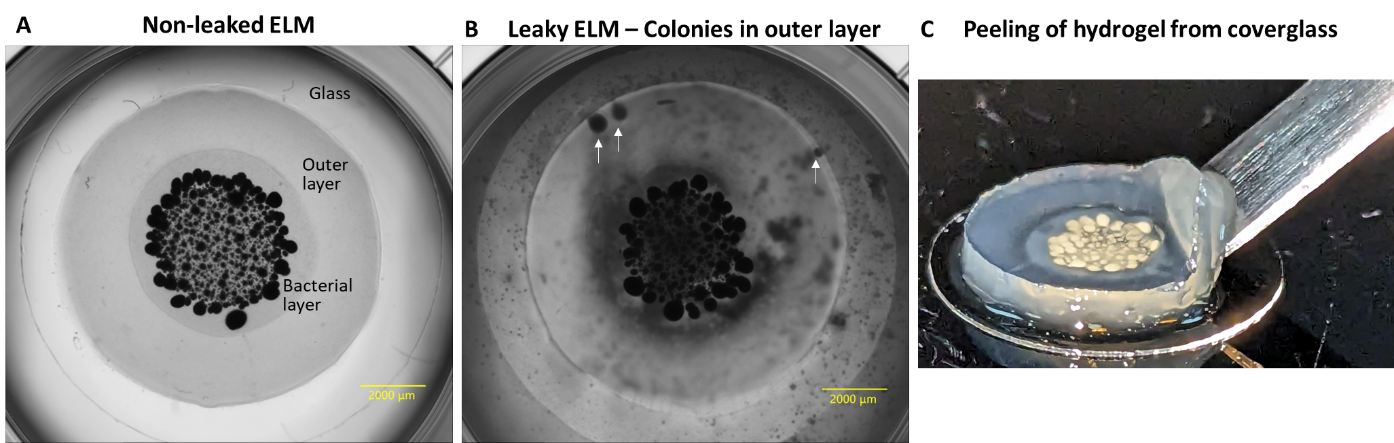


**Figure S6.** Brightfield microscopy images of 19-day old bilayer ELM films that were A. non-leaky and B. leaky. In B, the arrows point to bacterial colonies within the outer enveloping gel from which bacteria are considered to have grown out. C. Photo depicting the peeling off of the hydrogel from the coverglass due to improper bonding at the interface.
